# A semi-mechanistic population pharmacokinetic-pharmacodynamic model to assess downstream drug-target effects on erythropoiesis

**DOI:** 10.1007/s10928-025-09990-7

**Published:** 2025-07-24

**Authors:** S. Viktor Rognås, Franziska Schaedeli Stark, Maddalena Marchesi, Hanna E. Silber Baumann, João A. Abrantes

**Affiliations:** 1 Roche Pharma Research and Early Development, Pharmaceutical Sciences, Roche Innovation Center, Basel, Switzerland; 2 Department of Pharmacy, Uppsala University, Uppsala, Sweden

**Keywords:** Erythropoiesis, Hemoglobin synthesis, Blood kinetics, Pharmacometrics, Population modeling

## Abstract

Erythropoiesis is a complex process that results in the production of erythrocytes from hematopoietic stem cells in the bone marrow. This work aimed to develop a population pharmacokinetic-pharmacodynamic (PKPD) model describing erythropoiesis and hemoglobin synthesis following bitopertin, an inhibitor of glycine transporter 1 (GlyT1), administration. Data from a Phase 1 clinical trial in 67 healthy subjects administered bitopertin (10, 30, or 60 mg) or placebo for 120 days were analyzed. Hematological assessments included erythrocyte and reticulocyte counts, immature reticulocyte fraction, hemoglobin concentration, and mean corpuscular hemoglobin. The proposed semi-mechanistic model, which leverages data and physiological knowledge, was found to adequately simultaneously describe the dose- and time-dependent changes in the biomarkers. The framework was used to illustrate the potential outcome of hypothetical drug-target interactions at distinct stages of erythropoiesis and hemoglobin synthesis, exemplifying its usefulness in a clinical setting.

**Supplementary Information:**

The online version contains supplementary material available at 10.1007/s10928-025-09990-7.

## Introduction

Erythropoiesis is a critical and complex process responsible for generating hematopoietic stem cells in the bone marrow. These cells undergo several maturation stages and eventually develop into reticulocytes. Reticulocytes are released into the systemic blood circulation, where they mature into erythrocytes. This process is the cornerstone of human biology and is tightly regulated to maintain homeostasis in tissue oxygenation. Erythropoiesis is well understood, and intensive research has yielded several published mathematical models, ranging from empirical data-driven approaches to detailed mechanistic representations [1, 2] as well as extensive clinical pharmacokinetic-pharmacodynamic (PKPD) characterization of erythropoiesis-stimulating agents [3]. Erythropoietic parameter dynamics are important in both clinical and translational research and offer critical insights into various health conditions [4].

In drug development, a qualitative and quantitative understanding of the influence of investigational drugs on erythropoiesis is essential to ensure an appropriate balance between efficacy and safety. In silico models can help understand how a drug influences erythropoiesis, facilitate hypothesis testing or enable what-if scenario analysis for informed decisions. Semi-mechanistic population PKPD models describe the mechanistic understanding of a system (such as the interactions between various cell types, feedback mechanisms, and drug effects). These models rely on observed data while accounting for both physiological processes and time. They are suitable for simulations and characterizing the observed data’s central trends over time, variability between subjects, and residual variability [5].

Bitopertin is an inhibitor of glycine transporter 1 (GlyT1) previously developed for neurological indications [6, 7], demonstrating a mechanism-based inhibition of hemoglobin synthesis, resulting in reversible microcytic hypochromic anemia in rats [8]. An extensive Phase 1 study in healthy subjects was conducted to examine the long-term impact of bitopertin on the erythropoietic system in humans and to assess and mitigate the potential risk of developing anemia. Clinical data, including erythropoiesis-related markers, were collected during dosing and follow-up periods. Although bitopertin is no longer in development in the initially targeted indications, the generated hematological data remain valuable. These data can be leveraged to quantitatively describe the processes of erythropoietic and hemoglobin synthesis and the consequences of disrupting pathways within these systems [9].

The primary aim of this study was to develop a population PKPD model that describes erythropoiesis and hemoglobin synthesis after the administration of bitopertin. The same model was also designed to predict the downstream outcomes of hypothetical drug-target interactions at distinct stages of these processes.

## Methods

### Subjects and hematological biomarker data

The analysis included data from a Phase 1, multicenter, randomized, double-blind, placebo-controlled, parallel-group clinical trial. The primary aim of this study was to investigate the hematological and visual effects, safety, and tolerability of bitopertin. A total of 67 healthy males and females aged < 50 years were enrolled in the trial. The subjects were administered bitopertin (10, 30, or 60 mg) or placebo orally once daily for 120 days, followed by a 120-day follow-up period.

Blood samples for hematological assessments were collected at baseline, week 1, week 2, every 2 weeks thereafter until week 16, week 17 (at treatment end), week 18, and every 2 weeks thereafter until the end of the follow-up period at week 34. Additionally, rich pharmacokinetic (PK) sampling and records of individual bitopertin doses were available. These data enabled the estimation of individual PK parameters using a population PK model (data not shown).

### Development of the erythropoiesis model

#### Physiological process of erythropoiesis

The initial stages of erythrocyte development occur in the bone marrow, where various precursor cells are found. These precursor cells undergo a series of maturation stages for approximately 5 days [10, 11]. The most mature precursor form is the reticulocyte. Reticulocytes reside in the bone marrow for approximately 3 days and in the blood for approximately 1 day, leading to a total lifespan of approximately 4 days [4, 10]. Hemoglobin synthesis occurs in the early reticulocyte maturation stages, mainly in the bone marrow. Hemoglobin synthesis is confined to these immature reticulocytes because, after this stage, they no longer possess the necessary cellular machinery and ribosomal RNA for protein synthesis [4]. The reticulocyte matures into an erythrocyte by losing its internal organelles and remodeling its plasma membrane. Under hematologically normal conditions, erythrocytes remain stay in circulation for approximately 120 days, before being engulfed by splenic macrophages [10, 12].

#### Integration of available biomarkers into the model development

Early-stage precursors in the bone marrow were not directly observed because of the invasive nature of the sampling. Thus, this work focused on observations from blood samples, particularly reticulocytes, and their subdivision into mature and immature forms. Both the reticulocyte total blood count (RET) and the fraction of immature reticulocytes (IRF) in the blood are indicative of erythropoietic activity in the bone marrow [4].

Hemoglobin is a critical biomarker of the blood’s oxygen transportation capacity. A hemoglobin deficiency is used as a clinical marker for anemia [13]. Hemoglobin is assessed through total hemoglobin blood concentration (Hb_tot_) and mean corpuscular hemoglobin (MCH) in erythrocytes.

#### Structural model

A previously developed model that focused on hemoglobin turnover was used as a framework upon which a more extensive erythropoiesis model could be built [9]. The model describes erythrocyte turnover and the mean hemoglobin content within the erythrocytes as two parallel chains of four transit compartments that all share the same parameter for the transit rate constant. Hemoglobin turnover was described as the net effect of several processes. First, there was the synthesis of hemoglobin inside the precursor cells. Second, there was the turnover of erythrocytes in the blood. Finally, a feedback mechanism increases precursor recruitment when Hb_tot_ decreased. Bitopertin’s inhibitory effect on hemoglobin synthesis was modeled as an exposure-dependent decrease of the hemoglobin production rate. When erythrocytes with a reduced hemoglobin content enter the blood, Hb_tot_ consequently decreases, inducing a homeostatic feedback that stimulated the precursor recruitment rate.

The previously described model was expanded based on the following assumptions: reticulocyte maturation pathways described using a four-compartment structure representing immature and mature reticulocytes in bone marrow and blood [14]; an equal reticulocyte maturation rate in bone marrow and blood [4]; and homeostasis (steady state) at baseline. Furthermore, to reflect swift reticulocyte dynamics, an empirical tolerance mechanism was evaluated [15].

The model was fitted simultaneously to the observed data on RET, erythrocyte count (RBC), MCH, and IRF. Individual AUC_ss_ values were used as the exposure metric driving the bitopertin drug effect [9].

#### Statistical model

The need to include inter-individual variability (IIV) on a model parameter was evaluated. The IIV in a parameter was modeled using a log-normal distribution ${e}^{{\eta }_{i,p}}$. The random effect variable was assumed to follow a normal distribution ${\eta }_{i,p}\sim N\left(0,{\omega }_{p}^{2}\right)$ on parameter $p$ for individual $i$, where the variance ${\omega }_{p}^{2}$ was estimated. Correlations between variances ${\omega }_{p}^{2}$ were estimated in the later stages of model development.

The differences between the observed and individual model-predicted values were modeled as random quantities assumed to follow a normal distribution ${\epsilon }_{i,j,k}\sim N\left(0,{\sigma }_{k}^{2}\right)$ for individual $i$ and the observed value $j$ of the dependent variable (biomarker) $k$, where the residual unexplained variability (RUV) ${\sigma }_{k}^{2}$ was estimated. For each new dependent variable $k$ added to the model, a flexible combined additive and proportional residual error model was initially used and later challenged for reduction to either an additive or proportional error model.

#### Parameter estimation method

First-order conditional estimation (FOCE) with interaction was initially used, as it is relatively fast with rich data.^1^ When the model complexity increased, the Stochastic Approximation Expectation–Maximization (SAEM) estimation method was used because it handles complex PKPD models more efficiently [16]. In most models during development, SAEM was preceded by an exploratory Iterative Two-Stage (ITS) estimation step, followed by an Importance Sampling (IMP) evaluation step to evaluate the objective function value^2^ (OFV). When using SAEM, a full variance–covariance matrix was estimated ($OMEGA BLOCK), and parameters without inter-individual variability were fixed to have a 15% coefficient of variation ($OMEGA 0.0225 FIX), which allowed the SAEM algorithm to search the parameter space efficiently [16]. For SAEM, the AUTO = 1 option with 3000 burn-in iterations (NBURN = 3000) was typically used, followed by 1000 accumulation iterations (NITER = 1000). These settings were tweaked by visually inspecting the convergence of the OFV during the accumulation phase. If the OFV barely changed after a certain number of accumulations, NITER was reduced to a number just above that to save time. Mu-referencing was used whenever possible to achieve greater estimation efficiency [16]. The built-in convergence tester (from the AUTO = 1 option) for SAEM was used to determine when the burn-in phase statistically converged. Additionally, the SEED and RANMETHOD = 3S2P options were used to reduce stochastic noise and thus increase the reproducibility of both SAEM and IMP estimation methods.

The standard errors of the parameter estimates were obtained using the observed Fisher information matrix from the NONMEM covariance step ($COVARIANCE MATRIX = R).

#### Model qualification criteria

The selection of a candidate model was guided by scientific plausibility as well as numerical and graphical assessments [17].

The OFV was used for comparison between hierarchical models. A nominal p-value lower than 0.01 was chosen to be statistically significant for one degree of freedom—corresponding to a reduction in the OFV of more than 6.64 points. When comparing non-hierarchical models, the Akaike information criteria (AIC) was used [18].

The relative standard error^3^ (RSE) of an estimated parameter was also used as a model selection criterion. An RSE of < 40% for fixed effects and < 50% for random effects was considered adequate precision. The condition number was also evaluated, with a condition number above 1000 considered indicative of overparameterization.

An observation with a CWRES > 5 was considered an outlier. If an outlier was encountered, sensitivity analysis was performed to evaluate its influence on model parameters.

The ability of the population model to describe the data was assessed using prediction- and simulation-based diagnostics. Various population-based and individual-based goodness-of-fit (GOF) plots were used to evaluate the structural, RUV and IIV-model throughout assessment of model candidates [17]. Visual predictive checks (VPCs) were used to evaluate the predictive performance of the models through stochastic simulations. For all observations, 200 predictions were simulated, resulting in a distribution of predictions. The prediction distribution was then compared with the observations. Because rich data were available, a 95% prediction interval and a 95% confidence interval were used.

### Simulations

The developed model was used to stochastically simulate the consequences of an inhibitory drug-target effect of hypothetical compounds upon interaction with specific pathways in the erythropoiesis system. Each drug-target interaction defines a distinct mechanism occurring at different stages of erythrocyte development or hemoglobin synthesis. A treatment period of 120 days was assumed. The doses corresponded to either 0.5 or 2 times the AUC_50_ of bitopertin, where AUC_50_ is the area under the concentration–time curve associated with 50% of the maximal pathway inhibition (I_max_). The exposure levels translate to approximately 20% and 40% inhibition of the pathway (partial inhibition), respectively [9]. During this process, four potential drug-target effect mechanisms were examined. These mechanisms were:

- Mechanism A: Inhibition of hemoglobin synthesis (${R}_{\text{in},\;\text{PRE}}$), resembling the effect observed for bitopertin.
- Mechanism B: Inhibition of reticulocyte precursor production (${R}_{\text{in},\;\text{PRE}}$).
- Mechanism C: Inhibition of the differentiation of precursors to reticulocytes (${k}_{\text{PRE}}$).
- Mechanism D: Inhibition of hemoglobin synthesis (${R}_{\text{in},\;\text{MCH}}$) and full inhibition of Hb_tot_-driven feedback.

Additional information on the simulated mechanisms can be found in Supplementary A1.

### Software

Model development was performed using NONMEM (v7.4.3) [19], facilitated by PsN (v4.9.3) [20] and Improve (v2.5.1–5); additional simulations were performed in the mrgsolve R package (v1.0.3). For graphical analyses, R Statistical Software (v4.1.2) [21] was used, operating in the RStudio environment (v1.4.1717) [22], on a system running Windows 10 Enterprise.

## Results

### Data used for model development

Data from a total of 62 subjects receiving 10–60 mg of bitopertin or placebo were included in the model. Demographics and a summary of the available biomarker information are shown in Table 1.

**Table 1:** Summary of demographics and biomarker information at baseline, stratified by treatment arm

		All	0 mg	10 mg	30 mg	60 mg
Subjects	*n*	62	15	17	16	14
Demographics						
Males	*n* (%)	26 (42)	6 (40)	6 (35)	7 (44)	7 (50)
Females	*n* (%)	36 (58)	9 (60)	11 (65)	9 (56)	7 (50)
Age, years	mean	31.9	31.9	29.8	30.4	36.1
	SD	6.29	6.02	5.87	6.14	5.73
	median	31.0	29.0	31.0	29.0	36.5
	min	19.0	26.0	19.0	22.0	28.0
	max	45.0	44.0	40.0	44.0	45.0
Body weight, kg	mean	70.6	71.0	69.6	68.9	73.6
	SD	11.7	14.8	10.6	11.5	9.91
	median	69.0	68.6	68.0	68.7	72.2
	min	49.4	50.7	49.4	49.6	56.6
	max	108	108	89.0	86.3	91.0
Biomarkers at baseline						
RET, 10^9^/L	mean	40.2	42.2	43.1	34.7	40.7
	SD	13.9	13.6	18.8	10.0	10.1
	median	38.6	44.3	35.7	34.7	39.3
	min	17.7	22.3	17.7	21.1	26.9
	max	76.3	64.1	76.3	50.1	54.0
RBC, 10^12^/L	mean	4.70	4.66	4.71	4.71	4.72
	SD	0.412	0.430	0.407	0.476	0.358
	median	4.72	4.61	4.73	4.67	4.75
	min	3.87	3.87	3.94	3.94	4.01
	max	5.45	5.40	5.37	5.45	5.19
Hb_tot_, g/L^a^	mean	140.4	140.1	139.6	141.6	140.3
	SD	12.0	12.8	14.2	12.2	8.8
	median	142	142	133	141.5	141
	min	117	120	117	123	127
	max	165	158	165	163	153
MCH, pg	mean	29.9	30.1	29.7	30.2	29.8
	SD	1.40	1.59	1.57	0.917	1.50
	median	30.0	30.0	29.9	30.0	29.8
	min	26.0	27.0	26.1	29.0	26.0
	max	32.5	32.5	31.1	32.0	31.8
IRF	mean	0.052	0.052	0.052	0.045	0.059
	SD	0.027	0.028	0.037	0.017	0.023
	median	0.047	0.047	0.037	0.042	0.064
	min	0.006	0.012	0.006	0.021	0.017
	max	0.155	0.101	0.155	0.077	0.106

^a^Hb_tot_ data was not used to fit the final model

RET reticulocyte counts, RBC red blood cell (erythrocyte) counts, Hb_tot_ total blood hemoglobin concentration, MCH mean corpuscular hemoglobin, IRF immature reticulocyte fraction

**Fig. 1:**
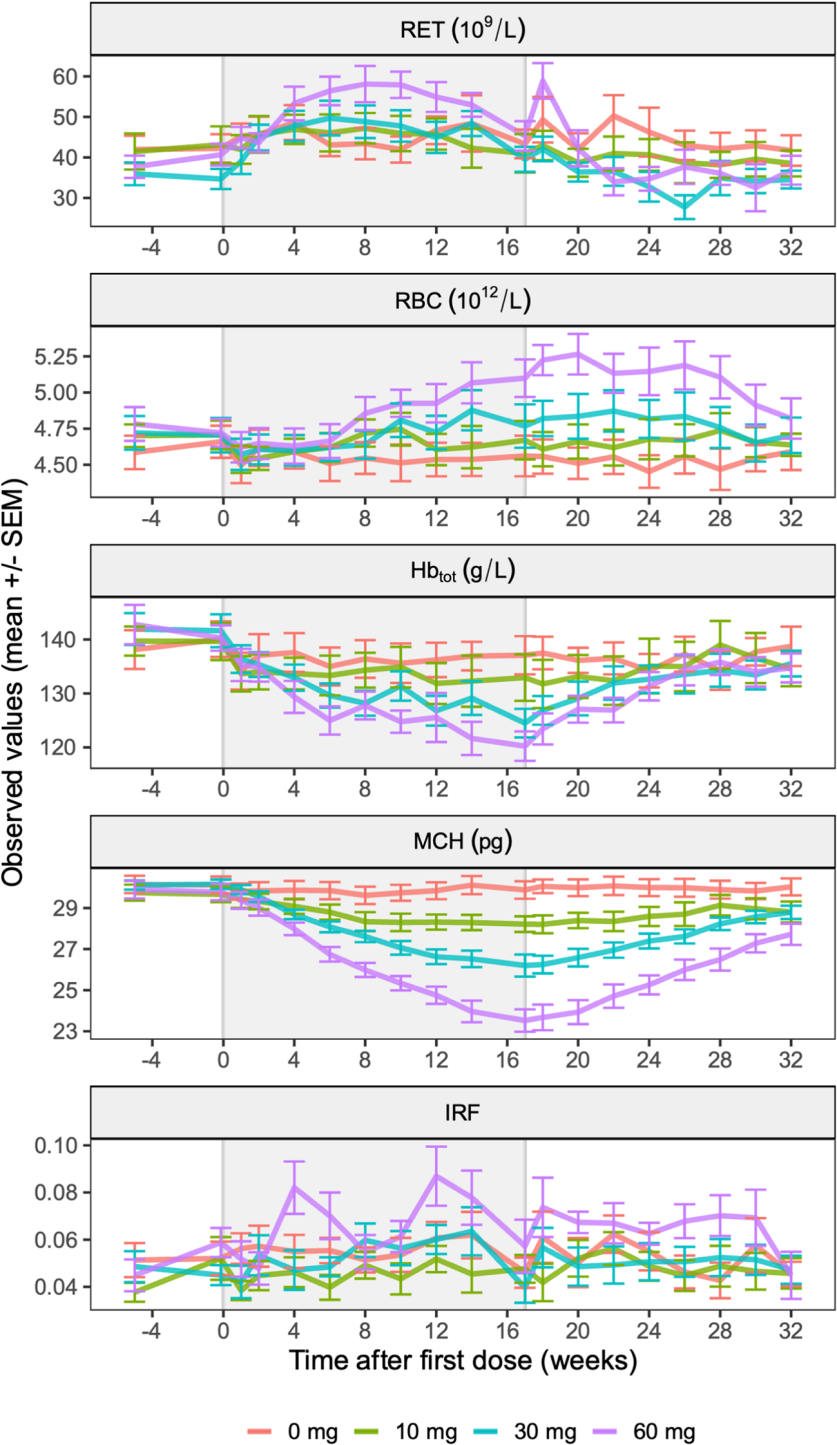
Observed hematological response data at baseline and during 4 months administration of 10, 30 or 60 mg bitopertin or placebo (shaded area), and 4 months follow-up. Data are represented as mean and standard errors (SEM) by treatment arm. RET reticulocyte counts, IRF immature reticulocyte fraction, RBC red blood cell (erythrocyte) counts, MCH mean corpuscular hemoglobin, Hb_tot_ total blood hemoglobin concentration

A visual display of the longitudinal data is provided in Fig. 1. Reticulocyte counts started increasing immediately after treatment initiation and reached a plateau after 4–6 weeks. Immature reticulocyte fraction values showed high variability and, as seen also in reticulocyte counts, the 60 mg cohort seemed to be associated with a larger magnitude of drug effect compared with other cohorts. Erythrocyte count, Hb_tot_, and MCH showed a dose-dependent change, which started immediately after the start of treatment for Hb_tot_ and MCH, but with a delay of 4–6 weeks for the erythrocyte count.

### Semi-mechanistic model structure

**Fig. 2:**
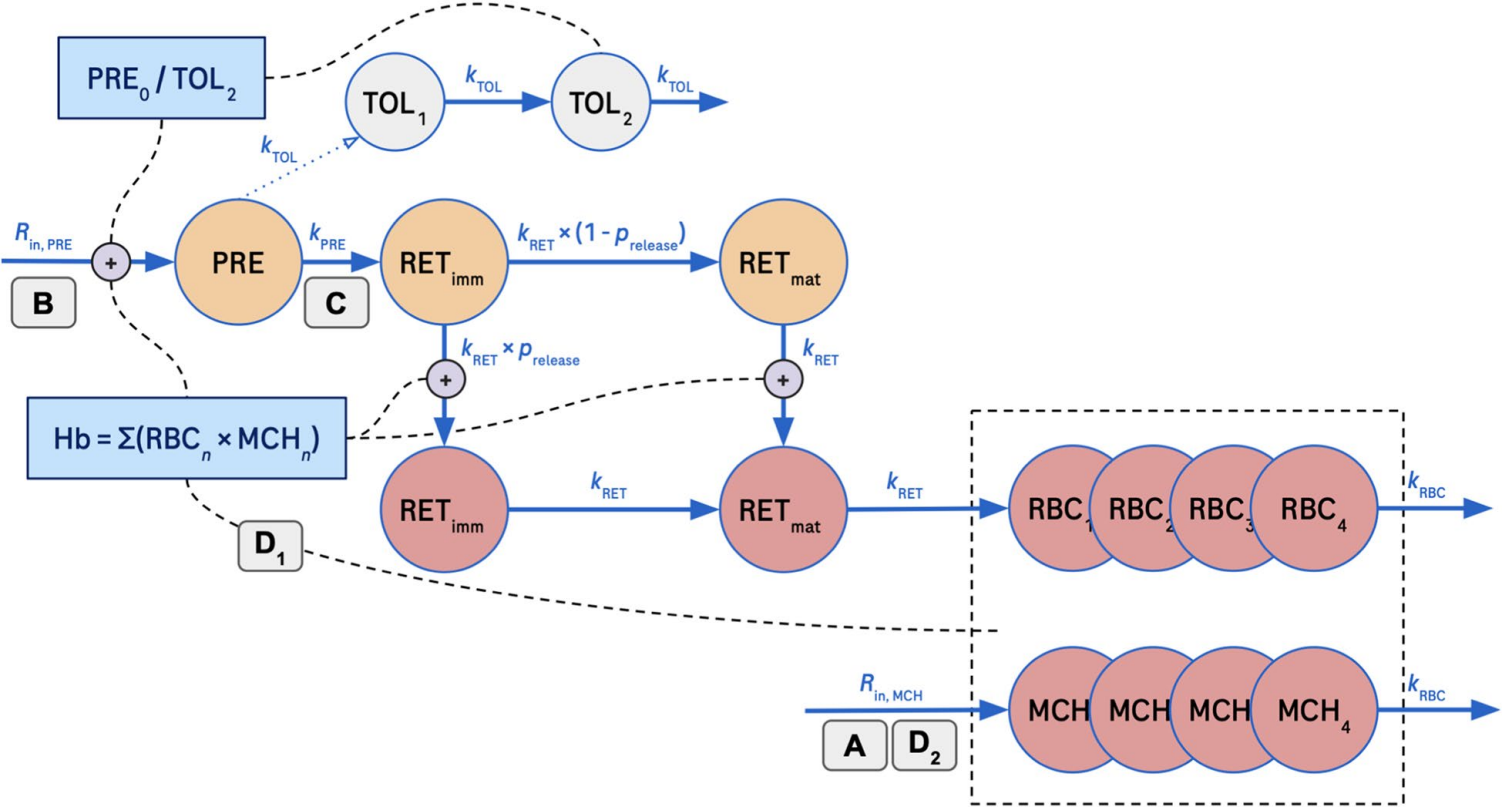
Graphical representation of the final model with rate constants. Orange color represents bone marrow compartments. Gray color represents empirical tolerance compartments. Red color represents the blood compartments. Blue solid lines represent cell transfer; the blue dotted line (from PRE to TOL_1_) represents no cell transfer; black dashed lines represent feedback and blue boxes calculation thereof. Bold letters (A, B, C, D_1_, D_2_) in a box represent a simulation mechanism. PRE precursors, PRE_0_ initial cell count in PRE compartment, TOL_*n*_ n^th^ tolerance compartment, RET_imm_ immature reticulocytes, RET_mat_ mature reticulocytes, RBC_*n*_ red blood cell (erythrocyte) count in the n^th^ compartment, MCH_*n*_ mean corpuscular hemoglobin in the n^th^ compartment, *R*_in, PRE_ input rate for precursors, *R*_in, MCH_ input rate for MCH, *k*_TOL_ rate constant for tolerance compartments, *k*_PRE_ rate constant for precursor compartment, *k*_RET_ rate constant for reticulocyte compartments, *k*_RBC_ rate constant for erythrocyte compartments, *p*_release_ probability of release for immature reticulocytes in bone marrow, Hb hemoglobin

A graphical illustration of the final model structure is shown in Fig. 2.

The model includes compartments representing blood, associated with observed data of reticulocyte count, erythrocyte count, MCH and IRF, and compartments representing unobserved data in the bone marrow. The process of erythropoiesis starts with precursor formation in the bone marrow. This compartment is characterized by a transit-time (LS_PRE_) that represents the lifespan of precursor cells in the bone marrow (Eq. 1):

1 $${\text{LS}}_{\text{PRE}}=\frac{1}{{k}_{\text{PRE}}}$$

Following the precursor compartment, there is a two-pathway, four-compartment structure that represents the reticulocyte maturation that may happen in the bone marrow or in the blood. This structure is parameterized using *k*_RET_, representing the first-order rate constant for reticulocyte maturation and migration from bone marrow into the circulation. The derivation of *k*_RET_ is shown in Eq. 2, where *k*_RBC_ is the first-order rate constant (see Eq. 3) for erythrocytes between the four transit-compartments (*n*_CTR_ = 4); RET_0_ is the baseline reticulocyte count, and IRF_0_ is the baseline immature reticulocyte fraction:

2 $${k}_{\text{RET}}=\frac{{k}_{\text{RBC}}\left(\frac{{\text{RBC}}_{0}}{{n}_{\text{CTR}}}\right)}{\left({\text{RET}}_{0}\left(1-{\text{IRF}}_{0}\right)\right)}$$

The fraction of reticulocytes being released prematurely into the blood is represented by the IRF, and is modeled by the parameter *p*_release_. Reticulocytes in the blood eventually develop into erythrocytes. The dynamics of erythrocytes are modeled by a four transit-compartment structure. These transit compartments, referred to as RBC_1–4_ in Fig. 2, exhibit equal transit times that cumulatively estimate the erythrocyte lifespan (LS_RBC_). The transit-rate constants for these compartments, *k*_RBC_, were calculated as the number of transit compartments (*n*_CTR_ = 4) divided by LS_RBC_:

3 $${k}_{\text{RBC}}=\frac{{n}_{\text{CTR}}}{{\text{LS}}_{\text{RBC}}}$$

In addition, a parallel transit-compartment chain, referred to as MCH_1–4_ in Fig. 2, represents the average hemoglobin content inside the erythrocytes in each of the compartments RBC_1-4_. Hb_tot_ was derived as the sum of the products across each transit compartment pair (Eq. 4).

4 $${\text{Hb}}_{\text{tot}}=\sum \limits_{n=1}^{4}{\text{RBC}}_{n}\times {\text{MCH}}_{n}$$

The model assumes no loss of hemoglobin within the erythrocyte during aging. Several modeling approaches were considered to account for the homeostatic systems feedback to maintain Hb_tot_ at constant levels. The feedback is induced by the fractional change from baseline of Hb_tot_ and it acts on the recruitment rate of hemoglobin-carrying cells to the blood. Initially, the feedback was included as a stimulation of the precursor rate in the bone marrow (*R*_in, PRE_). This model adequately described the observed dynamics in erythrocytes. By adding another feedback effect on *k*_RET_ to allow earlier and faster release of immature and mature reticulocytes from the bone marrow into blood circulation, the model fit to the reticulocyte count and IRF was further improved. An exponential relationship was superior to a linear stimulation function. Estimating the feedback components as two separate parameters did not significantly improve the fit and was numerically less stable.

To account for the fact that there is a reservoir of stem cells that can be depleted, and thus an initial increase in erythropoietic response that can decrease with time, a modulator mechanism was added using two tolerance compartments [15]. The recruitment of precursor cells (*R*_in_) is thus modulated by the current precursor amount relative to the initial amount.

#### Parameter estimates and model qualification

The parameter estimates, including fixed and random effects, along with their numerical precision are presented in Table 2.

**Table 2:** Parameter estimates and relative standard errors (RSE) for the final model parameters

Parameter	Definition	Estimate	Unit	%RSE	IIV (%CV)	Shrinkage^a^ (%)
LS_PRE_	Transit-time of precursor cells	5	days	Fixed		
LS_RBC_	Transit-time of red blood cells	125	days	4.44	28.02	16.92
RET_0_	Baseline reticulocyte count	39.80	10^9^/L	3.37	26.02	1.88
RBC_0, male_	Baseline RBC count for males	4.91	10^12^/L	0.78	5.34	1.63
RBC_diff, female_^a^	Difference in baseline RBC count for females	1.59	10^12^/L	3.18		
MCH_0_	Baseline mean hemoglobin content in RBC	29.80	pg	0.61	4.82	0.47
IRF_0_	Baseline immature reticulocyte fraction	4.71	%	4.31	32.09	3.88
Feedback^b^		2.42		8.84		
*k*_TOL_	Rate constant for tolerance compartments	0.022	day^−1^	12.86		
I_max,bitopertin_	Maximum bitopertin inhibition effect	0.6		Fixed		
AUC_50, bitopertin_	Bitopertin exposure where half of maximum inhibition is achieved	16.50	mg/L × h	8.91	49.50	24.97
ε_RET, proportional_	Proportional residual error for RET	0.22		2.35		
ε_RBC, additive_	Additive residual error for RBC	0.18	10^12^/L	2.22		
ε_MCH, additive_	Additive residual error for MCH	0.35	pg	2.30		
ε_IRF, additive_	Additive residual error for IRF	0.96	%	16.48		
ε_IRF, proportional_	Proportional residual error for IRF	0.38		5.41		

^a^RBC_diff, female_ = RBC_0, male_—RBC_0, female_

^b^The feedback was implemented as follows: ${\text{Stimulation} = e}^{\frac{{\text{Hb}}_{\text{tot}}-{\text{Hb}}_{0}}{{\text{Hb}}_{0}} \times\;\text{Feedback}}$. The condition number was 272

All parameters were estimated with adequate precision (Table 2). Shrinkage values for IIV were all below 25%, indicating well-informed individual parameters. The I_max, bitopertin_ value was fixed to a previously estimated value to increase model stability. This value agreed with the pre-clinical knock-out mice results [23].

The typical lifespan of an erythrocyte (LS_RBC_) was estimated to be 125 days. This duration corresponds to the time between the appearance of the erythrocyte in the blood to its removal from circulation. The transit time of the precursors was fixed at a literature value of 5 days [10, 11]. The typical time a reticulocyte resided in the blood at baseline was 20 h (IRF_0_ × (2 × LS_RET_) + (1−IRF_0_) × LS_RET_), where LS_RET_ is defined similarly to LS_PRE_ (Eq. 1). The typical time it takes a reticulocyte to go from an immature stage in the bone marrow to a circulating erythrocyte, was approximately 2.4 days (3 × LS_RET_).

**Fig. 3:**
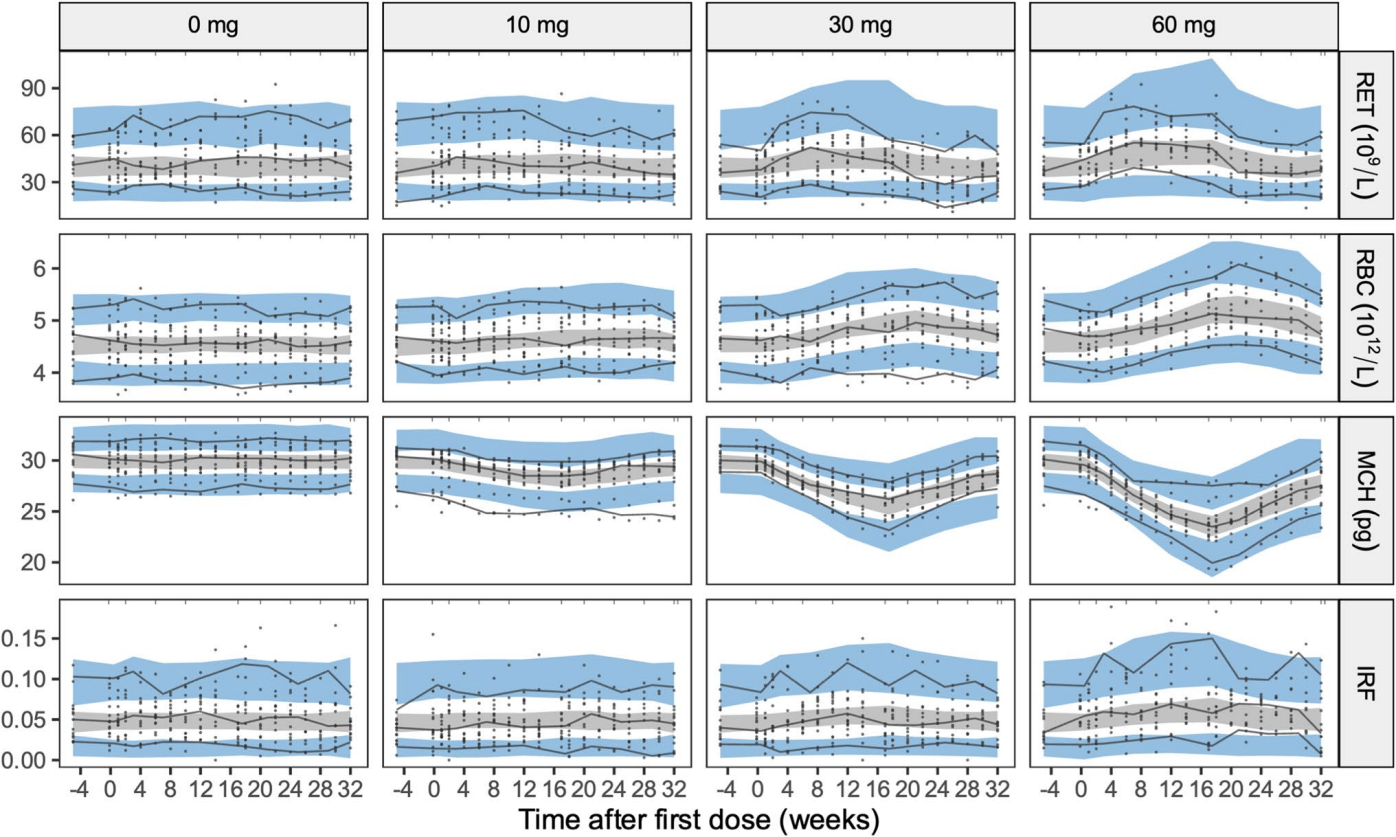
Visual predictive check by dose for the four hematological response variables (RET, RBC, MCH, and IRF). Black solid lines represent the 5th, 50th, and 95th percentiles of the observations. Shaded areas represent 90% confidence intervals of the 5th, 95th (blue), and 50th (gray) simulated percentiles from 200 simulated datasets

Overall, the model was found to describe the observed data adequately. The VPC shows no marked bias (Fig. 3), confirming that the general trends in the longitudinal data and variability are well captured by the model. Reticulocyte counts exhibit a slight bias, which tends to increase with increasing doses. However, this was deemed to have a limited impact in the ability of the model to describe the erythrocyte count data.

**Fig. 4:**
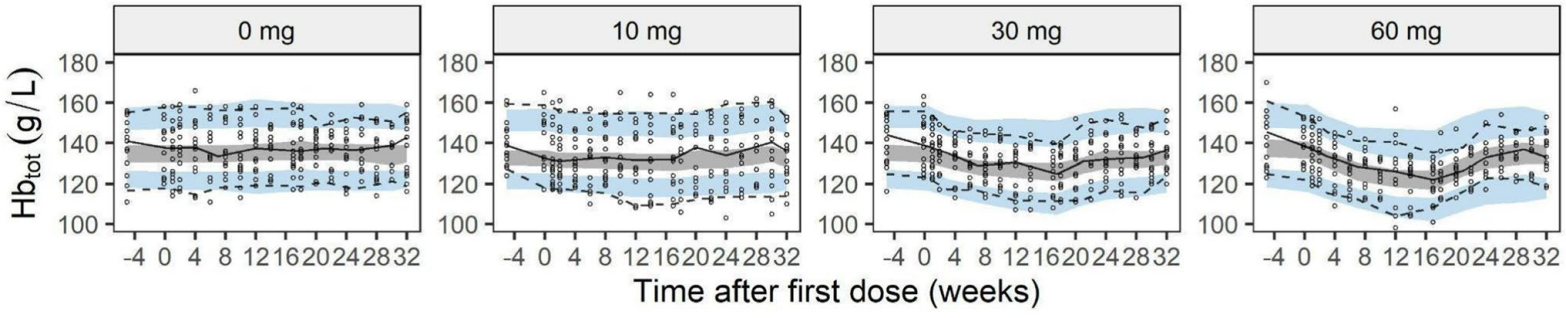
Visual predictive check by dose for observed total hemoglobin blood concentration. Black solid lines represent the 5th, 50th, and 95th percentiles of the observations. Shaded areas represent 90% confidence intervals of the 5th, 95th (blue), and 50th (gray) simulated percentiles from 200 simulated datasets

A comparison of the model-predicted Hb_tot_ and the observed Hb_tot_ values (not included in the model) is shown in Fig. 4. The observed trends in the Hb_tot_ data were well predicted by the model, even though Hb_tot_ observations were not included during model development.

### Simulations of hypothetical drug-target interactions

**Fig. 5:**
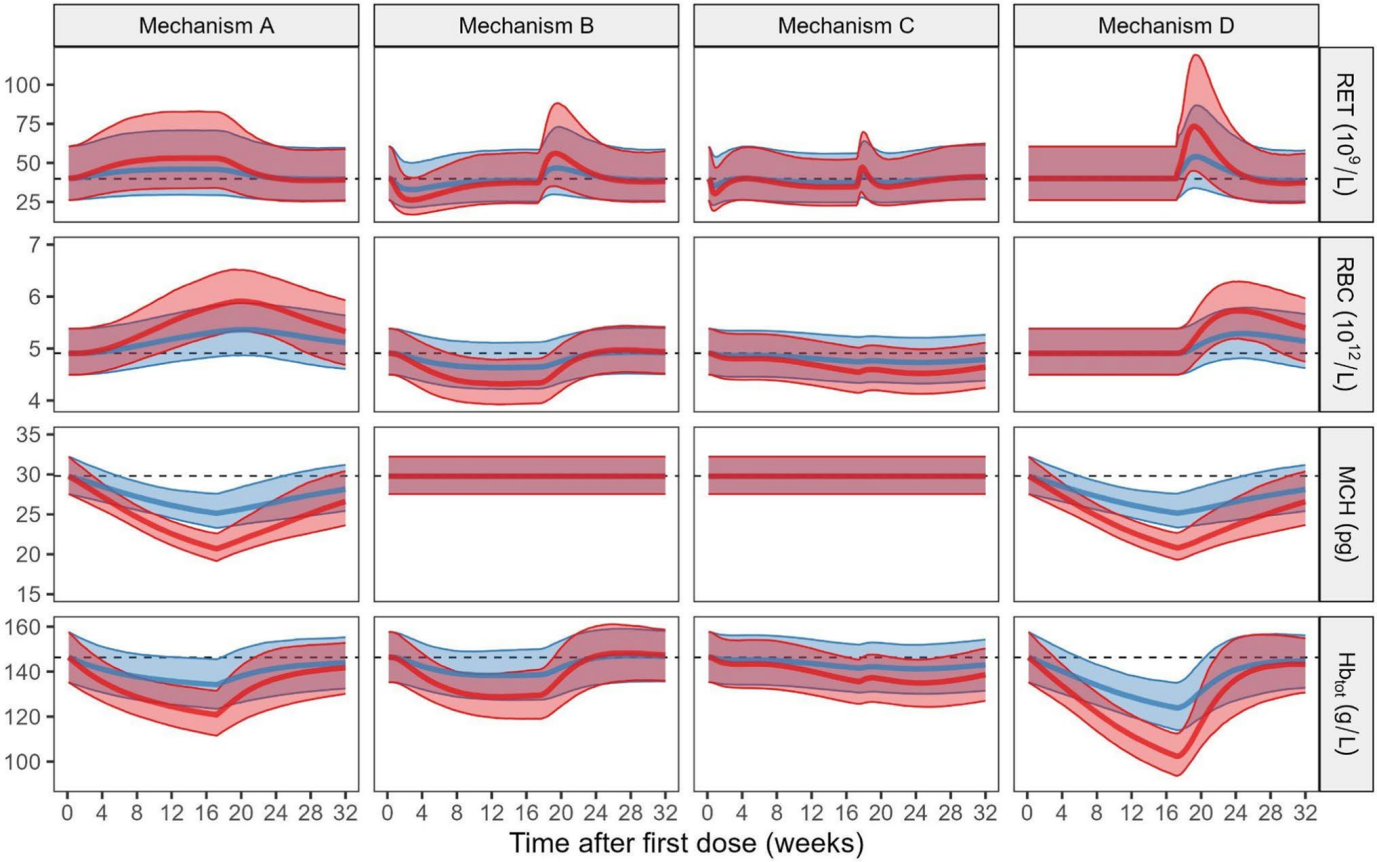
Simulated outcomes of biomarkers from hypothetical drug-target interactions following 120 days of treatment (dose corresponding to 0.5 × AUC_50_ in blue, and to 2 × AUC_50_ in red) and 120 days of follow-up in 2000 male subjects. For each dose-level, the solid line represents the median, and the shaded area the 90% prediction interval which reflects inter-individual variability. The horizontal dashed line represents the median baseline in the absence of drug effect

An illustration of the model predictions in the presence of hypothetical drug-target interaction mechanisms (marked A–D) is shown in Fig. 5.

#### Mechanism A: inhibition of hemoglobin synthesis rate

Reticulocyte count was characterized by progression to a new equilibrium, returning to baseline swiftly after treatment cessation. A similar phenomenon can be observed at different rates for erythrocytes and MCH but does not reach a steady state. A gradual decline was seen for Hb_tot_ that later returned to the pre-treatment baseline post-cessation.

#### Mechanism B: Inhibition of reticulocyte early precursor recruitment rate

This mechanism caused an initial descent in reticulocyte count during the first two weeks, followed by a recovery phase. Reticulocyte counts returned to baseline approximately 90 days after start of treatment. Following cessation of treatment at day 120, the reticulocyte count increased rapidly over the next two weeks, eventually stabilizing at baseline levels by day 180 (60 days after end of treatment). Erythrocytes showed a decline, reaching a new equilibrium at approximately 60 days after treatment initiation. After treatment cessation at day 120, erythrocyte counts rapidly returned to baseline levels within approximately 30 days. MCH remained unaffected. Consequently, hemoglobin dynamics aligned with the changes in erythrocyte count.

#### Mechanism C: Inhibition of reticulocyte late precursor maturation rate

Similar to Mechanism B, but at a lesser magnitude, this mechanism showed a rapid initial decline in reticulocyte count, later stabilizing slightly below the pre-treatment levels. The erythrocyte trajectory consequently showed a gradual decline after the 70-day mark because of the reduced influx of reticulocytes, a trend that continued beyond the cessation of treatment. The trend in Hb_tot_ was parallel to the erythrocyte count, given the unaffected state of MCH.

#### Mechanism D: Simultaneous inhibition of hemoglobin synthesis rate and full inhibition of homeostatic feedback affecting reticulocyte early precursor recruitment rate

Mechanism D consists of two parts. Mechanism D_1_ fully inhibits the hemoglobin-driven feedback affecting reticulocyte early precursor recruitment rate, and Mechanism D_2_ inhibits the production of hemoglobin. This combined mechanism resulted in steady levels of reticulocyte counts and erythrocytes during the drug effect period whereas the MCH and hemoglobin showed a continuous decline. When treatment was stopped, reticulocyte counts and erythrocytes depict a brief spike due to the re-activation of the hemoglobin-driven feedback before gradually returning to their respective pre-treatment baselines. Simultaneously, MCH and hemoglobin returned to baseline due to complete reactivation of hemoglobin synthesis and homeostatic feedback.

## Discussion

In this work, we developed a semi-mechanistic PKPD model to allow for the prediction of effects on erythropoiesis from various medical scenarios and drug-target interactions. The model combines a data-driven approach with the mechanistic understanding of erythropoiesis and hemoglobin synthesis. We expanded a previously developed model that focused on blood hemoglobin turnover to predict the risk of anemia under long-term bitopertin treatment in the originally targeted neurological indications [9]. Integrating short-term biomarker data, such as reticulocytes and the fraction of immature cells released into the blood, makes this model more versatile. The model adequately predicted the risk of anemia by including the erythrocyte-count and MCH data.

The model was developed on a rich hematological dataset collected in a placebo-controlled clinical Phase 1 study involving bitopertin in healthy subjects. This dataset is an invaluable source of information for studying the consequences of partial inhibition of hemoglobin synthesis in erythrocyte precursors through bitopertin administration for an entire erythrocyte lifespan (~ 120 days). The longitudinal dataset is particularly suitable to propose a semi-mechanistic compartmental model describing several aspects of the human erythropoiesis and hemoglobin homeostasis system in a quantitative manner.

The observed hematological response data corroborate that bitopertin inhibits hemoglobin synthesis, as seen in a gradual dose-dependent decrease in MCH during treatment. This suggests that older erythrocytes are gradually replaced by younger cells with lower hemoglobin content, a result of bitopertin’s effect, which ultimately lowers the MCH. The effect on Hb_tot_ is the combined result of changes in both MCH and erythrocyte count (Fig. 2), making it less pronounced than the effect on MCH alone. While cell count and hemoglobin data do not necessarily offer a full picture of erythropoiesis, changes in these parameters would prompt the body to adjust, ensuring sufficient oxygen delivery to the tissues. This was reflected in the increase of the number of circulating reticulocytes as a homeostatic response to decreased Hb_tot_.

As observed for reticulocyte counts, IRF appears to increase with increasing doses of bitopertin. An increase in IRF indicates higher reticulocyte production and accelerated release of immature reticulocytes from the bone marrow. However, IRF data are more variable and more responsive to short-term signaling of the homeostatic system response, which might be difficult to capture in the data from 2- to 4-weekly sampling. The elevated production of reticulocytes results in an observed increase in erythrocytes, although delayed, owing to the substantially higher number of circulating erythrocytes (reticulocyte count constitutes only 1–2% of erythrocyte count) and their slower turnover rate.

Despite the treatment duration covering approximately one erythrocyte lifespan, the observed erythrocyte count does not appear to completely reach a new steady state, as would be expected. This is thought to be due to the feedback which leads to a shift in the time and magnitude of the new steady state of Hb_tot_ and erythrocyte count.

The final model is a transit-compartment model representing the different maturation stages, cell migration and hemoglobin pathways during erythropoiesis [9, 14, 24, 25]. This type of model, a special case of a lifespan-based indirect response model, accurately approximates the age distribution of erythrocytes [26, 27]. The estimated typical lifespan of erythrocytes in our model was 125 days, which is comparable to the literature values [10].

The model assumes that the release rate of reticulocytes from the bone marrow is the same regardless of the maturation stage, and the maturation rate for reticulocytes is the same in bone marrow and blood. Of note, the time for a reticulocyte to mature in the bone marrow and differentiate into an erythrocyte was calculated to be ~ 2.4 days, which was slightly shorter than 3.8 days reported in the literature [10]. To maintain homeostasis of Hb_tot_, the final model incorporates a feedback mechanism that resembles the effect of endogenous erythropoietin production in response to a decrease in hemoglobin concentration during treatment [28]. Additionally, the model includes an empirical tolerance term to account for the relative depletion of the stem cell reservoir. The predictive performance was adequate for all hematological biomarkers. The data supports the model assumptions, as shown by the model fit and precise parameter estimates.

To demonstrate the practical application of the modeling framework, we used the model to simulate the inhibitory drug-target effects, assuming a constant 120-day inhibition when interacting with specific pathways in the erythropoiesis system (Mechanisms A–D). Whereas Mechanism A resembles the mechanism of bitopertin, which inhibits hemoglobin synthesis, the remaining mechanisms represent the effect of hypothetical compounds. A transient increase in reticulocytes was observed following treatment cessation in Mechanisms B, C, and D. This rebound arises from the delayed feedback regulation of erythropoiesis. Since hemoglobin-mediated feedback acts later in the process, suppression of reticulocyte production lags behind after treatment stops, leading to a temporary elevation in circulating reticulocytes before equilibrium is restored.

This framework can be used to optimize dose selection during clinical drug development, or for hypothesis generation and testing of mechanisms of action of compounds with an effect on the erythropoiesis system. Only in Mechanism D, simulations of Hb_tot_ fell below 100 g/L, the threshold for moderate anemia (CTCAE Grade 2), in a subset of simulated individuals; however, the magnitude of the effect depends on the assumed inhibitory potency. Mechanism B (inhibition of reticulocyte precursor production) suggested that Hb_tot_ would stabilize at a new lower steady state within 60 days of the effect. In contrast, Mechanisms A and D showed a continuous decline in Hb_tot_, indicating potential long-term effects. The presented framework could also be adjusted for application in a disease-modeling context to quantify the impact of a certain medical condition on erythropoiesis. For instance: Mechanism B could be present following a transient aplastic crisis caused by a Parvovirus B19 infection that targets erythroid progenitor cells in the bone marrow, leading to a sharp decline in erythrocyte and reticulocyte counts, and Hb_tot_, while MCH remains constant; Mechanism C could mimic the signals seen in aplastic anemia where the bone marrow fails to produce mature blood cells leading to a reduced output of reticulocytes and a decline in erythrocytes and hemoglobin, and; Mechanism D could be present in an anemia of chronic disease, where inflammation simultaneously impairs hemoglobin synthesis and suppresses the bone marrow's compensatory response. Additionally, the model can be updated with new data and added modules during learn-confirm cycles.

A limitation of the current work is that there was no external assessment with data from other compounds. However, the estimated parameters are in agreement with physiological literature values, providing some degree of external scientific validity. Despite this, it would be valuable to further qualify the model with other study data, such as data from compounds with different MoAs. Of note, it is important to acknowledge that our model does not explicitly incorporate the dynamics of iron metabolism, a key regulator of erythropoiesis. The mechanism of action of bitopertin, which impacts the availability of glycine necessary for heme production, is distinct from iron-restricted erythropoiesis. While both pathways can result in reduced hemoglobin content, the latter is characterized by ineffective erythropoiesis and suppressed cell production. In contrast, the effects observed in our study and captured by the model show a compensatory increase in reticulocyte and erythrocyte counts in response to declining hemoglobin levels. Additionally, iron levels increased with bitopertin treatment, further suggesting that glycine availability, not iron, was the limiting factor. Another limitation is the age restriction to participants under 50 years which excludes data from older populations, who may respond differently to the treatment. Therefore, it would also be valuable to fit the model to larger datasets, for instance, with a more heterogeneous patient population in order to characterize the variability and effect of covariates more extensively. Of note, certain components have a partly empirical nature, such as the hemoglobin-driven feedback and tolerance mechanisms with their associated parameter estimates, which should be carefully assessed when using the model for extrapolation scenarios.

As highlighted previously, this model can be applied to a wide range of possible drug-target effects. The model can easily be extended with additional modules based on specific or evolving research interests. An example of such an extension would be the implementation of the reticulocyte average hemoglobin content (CHr), a short-term biomarker that reflects the functional iron availability during the short lifespan of reticulocytes [4]. Exploring the interactions between these biomarkers could further enrich the model’s predictive power. Yet another extension could be the incorporation of blood loss by sampling, which although it was tested in this work, was found to be statistically non-significant and therefore not retained in the model [29]. Conversely, the model could also be adapted to account for blood transfusions, which is essentially the physiological opposite of blood loss, representing an external input of mature red blood cells, which may be relevant in clinical scenarios such as anemia management or supportive care in hematological disorders.

## Conclusions

An integrated semi-mechanistic population pharmacokinetic-pharmacodynamic model was developed to describe the processes of erythropoiesis and hemoglobin synthesis. Simulations of hypothetical drug-target effects on different stages of erythropoiesis and hemoglobin production illustrated the influence of the assumed mechanism of action of compounds on the hematological response.

Combining this integrated model framework with mechanistic understanding from other compounds can be used to inform decision-making in drug development. Our work offers a data-driven, physiologically-backed model that may aid further innovations in the field, steering us closer to a comprehensive understanding of red blood cell dynamics and its implications in clinical and translational research. It thus helps to fill a critical gap in assessing the effects on long-term biomarkers (i.e., Hb_tot_ and erythrocyte count) downstream of the treatment interaction.

## Supplementary Information

Below is the link to the electronic supplementary material.

Supplementary file1 (DOCX 50 KB)

## Data Availability

No datasets were generated or analysed during the current study.

## Footnotes

1 The number of observations per subject exceeds the number of estimated random effect parameters, and the data covers the full concentration-time profile.

2 Proportional to minus two times the log-likelihood (-2 log ℒ) of the data.

3 RSE = point estimate/standard error.

## References

[1] Lledó-García R, Kalicki RM, Uehlinger DE, Karlsson MO (2012) Modeling of red blood cell life-spans in hematologically normal populations. J Pharmacokinet Pharmacodyn 39:453–462. 10.1007/s10928-012-9261-5 22847734 10.1007/s10928-012-9261-5

[2] Shrestha RP, Horowitz J, Hollot CV et al (2016) Models for the red blood cell lifespan. J Pharmacokinet Pharmacodyn 43:259–274. 10.1007/s10928-016-9470-4 27039311 10.1007/s10928-016-9470-4 PMC4887310

[3] Doshi S, Krzyzanski W, Yue S et al (2013) Clinical pharmacokinetics and pharmacodynamics of erythropoiesis-stimulating agents. Clin Pharmacokinet 52:1063–1083. 10.1007/s40262-013-0098-x 23912564 10.1007/s40262-013-0098-x

[4] Piva E, Brugnara C, Spolaore F, Plebani M (2015) Clinical utility of reticulocyte parameters. Clin Lab Med 35:133–163. 10.1016/j.cll.2014.10.004 25676377 10.1016/j.cll.2014.10.004

[5] Mould DR, Upton RN (2012) Basic concepts in population modeling, simulation, and model-based drug development. CPT Pharmacomet Syst Pharmacol 1:e6. 10.1038/psp.2012.4 10.1038/psp.2012.4 PMC3606044 23835886

[6] Bugarski-Kirola D, Iwata N, Sameljak S et al (2016) Efficacy and safety of adjunctive bitopertin versus placebo in patients with suboptimally controlled symptoms of schizophrenia treated with antipsychotics: results from three phase 3, randomised, double-blind, parallel-group, placebo-controlled, multicentre studies in the SearchLyte clinical trial programme. Lancet Psychiatry 3:1115–1128. 10.1016/S2215-0366(16)30344-3 27816567 10.1016/S2215-0366(16)30344-3

[7] Bugarski-Kirola D, Blaettler T, Arango C et al (2017) Bitopertin in negative symptoms of schizophrenia—results from the Phase III FlashLyte and DayLyte Studies. Biol Psychiatry 82:8–16. 10.1016/j.biopsych.2016.11.014 28117049 10.1016/j.biopsych.2016.11.014

[8] Winter M, Funk J, Körner A et al (2016) Effects of GlyT1 inhibition on erythropoiesis and iron homeostasis in rats. Exp Hematol 44:964-974.e4. 10.1016/j.exphem.2016.07.003 27403535 10.1016/j.exphem.2016.07.003

[9] Schaedeli Stark F, Martin-Facklam M, Hofmann C et al. (2012) Semi-physiologic population PKPD model characterizing the effect of bitopertin (RG1678) glycine reuptake inhibitor on hemoglobin turnover in humans

[10] Finch CA, Harker LA, Cook JD (1977) Kinetics of the formed elements of human blood. Blood 50:699–707. 10.1182/blood.V50.4.699.699 332255

[11] Koury MJ, Koury ST, Kopsombut P, Bondurant MC (2005) In vitro maturation of nascent reticulocytes to erythrocytes. Blood 105:2168–2174. 10.1182/blood-2004-02-0616 15528310 10.1182/blood-2004-02-0616

[12] Thiagarajan P, Parker CJ, Prchal JT (2021) How do red blood cells die? Front Physiol 12:655393. 10.3389/fphys.2021.655393 33790808 10.3389/fphys.2021.655393 PMC8006275

[13] U.S. Department of Health and Human Services (2017) Common Terminology Criteria for Adverse Events (CTCAE) Version 5.0. https://ctep.cancer.gov/protocoldevelopment/electronic_applications/ctc.htm#ctc_50

[14] Thorsted A, Zecchin C, Berges A et al (2024) Predicting the long-term effects of therapeutic neutralization of oncostatin M on human hematopoiesis. Clin Pharmacol Ther. 10.1002/cpt.3246 38501358 10.1002/cpt.3246

[15] Gabrielsson J, Hjorth S (2016) Pattern recognition in pharmacodynamic data analysis. AAPS J 18:64–91. 10.1208/s12248-015-9842-5 26542613 10.1208/s12248-015-9842-5 PMC7583549

[16] Bauer RJ (2019) NONMEM Tutorial Part II: Estimation Methods and Advanced Examples. CPT Pharmacomet Syst Pharmacol psp4.12422. 10.1002/psp4.12422 10.1002/psp4.12422 PMC6709422 31044558

[17] Nguyen THT, Mouksassi M, Holford N et al (2017) Model evaluation of continuous data pharmacometric models: metrics and graphics. CPT Pharmacomet Syst Pharmacol 6:87–109. 10.1002/psp4.12161 10.1002/psp4.12161 PMC5321813 27884052

[18] Akaike H (1974) A new look at the statistical model identification. IEEE Trans Autom Control 19:716–723. 10.1109/TAC.1974.1100705

[19] Bauer RJ (2019) NONMEM tutorial part I: Description of commands and options, with simple examples of population analysis. CPT Pharmacomet Syst Pharmacol psp4.12404. 10.1002/psp4.12404 10.1002/psp4.12404 PMC6709426 31056834

[20] Lindbom L, Ribbing J, Jonsson EN (2004) Perl-speaks-NONMEM (PsN)—a Perl module for NONMEM related programming. Comput Methods Programs Biomed 75:85–94. 10.1016/j.cmpb.2003.11.003 15212851 10.1016/j.cmpb.2003.11.003

[21] R Core Team (2021) R: A language and environment for statistical computing. Austria, Vienna

[22] Posit team (2023) RStudio: Integrated development environment for R. Posit Software, PBC, Boston, MA

[23] Garcia-Santos D, Schranzhofer M, Bergeron R et al (2017) Extracellular glycine is necessary for optimal hemoglobinization of erythroid cells. Haematologica 102:1314–1323. 10.3324/haematol.2016.155671 28495915 10.3324/haematol.2016.155671 PMC5541866

[24] Pérez-Ruixo JJ, Krzyzanski W, Bouman-Thio E et al (2009) Pharmacokinetics and pharmacodynamics of the erythropoietin Mimetibody™ Construct CNTO 528 in healthy subjects. Clin Pharmacokinet 48:601–613. 10.2165/11317190-000000000-00000 19725594 10.2165/11317190-000000000-00000

[25] Doshi S, Chow A, Pérez-Ruixo JJ (2010) Exposure-response modeling of darbepoetin alfa in anemic patients with chronic kidney disease not receiving dialysis. J Clin Pharmacol 50:75S-90S. 10.1177/0091270010377201 20881221 10.1177/0091270010377201

[26] Hamrén B, Björk E, Sunzel M, Karlsson M (2008) Models for plasma glucose, HbA1c, and hemoglobin interrelationships in patients with type 2 diabetes Following Tesaglitazar Treatment. Clin Pharmacol Ther 84:228–235. 10.1038/clpt.2008.2 18388881 10.1038/clpt.2008.2

[27] Krzyzanski W (2011) Interpretation of transit compartments pharmacodynamic models as lifespan based indirect response models. J Pharmacokinet Pharmacodyn 38:179–204. 10.1007/s10928-010-9183-z 21107661 10.1007/s10928-010-9183-z PMC3177953

[28] Pérez-Ruixo JJ, Krzyzanski W, Hing J (2008) Pharmacodynamic analysis of recombinant human erythropoietin effect on reticulocyte production rate and age distribution in healthy subjects. Clin Pharmacokinet 47:399–415. 10.2165/00003088-200847060-00004 18479174 10.2165/00003088-200847060-00004 PMC3145321

[29] Overgaard RV, Karlsson M, Ingwersen SH (2007) Pharmacodynamic model of interleukin-21 effects on red blood cells in cynomolgus monkeys. J Pharmacokinet Pharmacodyn 34:559–574. 10.1007/s10928-007-9059-z 17516151 10.1007/s10928-007-9059-z

