# Supplementary material for: A semi-mechanistic population pharmacokinetic-pharmacodynamic model to assess downstream drug-target effects on erythropoiesis
Source: J Pharmacokinet Pharmacodyn. 2025 Jul 24;52(4):42. doi: 10.1007/s10928-025-09990-7 (PMC12289712; doi:10.1007/s10928-025-09990-7)
Supplement: Supplementary file 1 — Supplementary file1 (DOCX 50 KB) [file 10928_2025_9990_MOESM1_ESM.docx]

# Appendix

## 1. Definitions

| **Table 3** Definitions | | | |
| --- | --- | --- | --- |
| **Entity** | **Abbreviation** | **Unit** | **Definition** |
| Total reticulocyte blood count | RET | 10^6^ /L | ${RET}_{immature}+{RET}_{mature}$ |
| Cellular hemoglobin in reticulocytes | CHr | pg | $CHr\equiv{Hb}_{RET}/{RET}_{tot}$ |
| Total erythrocyte (red blood cell) blood count | RBC_tot_ | 10^9^ /L | $\sum_{n=1}^{4} {RBC}_{n}$ |
| Total hemoglobin blood concentration | Hb_tot_ | g/L | ${MCH}_{RBC}\times\sum_{n=1}^{4} {RBC}_{n}$ |
| Mean corpuscular hemoglobin | MCH | pg | ${MCH}_{RBC}={Hb}_{RBC} /\sum_{n=1}^{4} {RBC}_{n}$ |
| Immature reticulocyte fraction | IRF | 1 | ${RET}_{immature}/{RET}_{tot}$ |
| Cellular hemoglobin in reticulocytes | CHr | pg | $CHr\equiv{Hb}_{RET}/{RET}_{tot}$ |

## 2. Differential equation system of the model

| $\frac{dPRE}{dt}=R_{in, PRE}\times\xi\times\frac{{PRE}_{0}}{{TOL}_{2}}-k_{LS, PRE}\times PRE$ | (1) |
| --- | --- |
| $\frac{d{RET}_{imm, bm}}{dt}=k_{LS,PRE}\times PRE-k_{LS, PRE}\times\xi\left( {RET}_{imm, bm}\times p_{release} \right)-k_{LS, RET} \left( {RET}_{imm, bm} \left( 1-p_{release} \right) \right)$ | (2) |
| $\frac{d{RET}_{mat, bm}}{dt}=k_{LS, RET} \left( {RET}_{imm, bm} \left( 1-p_{release} \right) \right)-k_{LS, RET}\times\xi\times{RET}_{mat, bm}$ | (3) |
| $\frac{d{RET}_{imm, bl}}{dt}=k_{LS, RET}\times\xi\left( {RET}_{imm, bm}\times p_{release} \right)-k_{LS, RET}\times{RET}_{imm, bl}$ | (4) |
| $\frac{d{RET}_{mat, bl}}{dt}=k_{LS, RET}\times\xi\times{RET}_{mat, bm}+k_{LS, RET}\times{RET}_{imm, bl}-k_{LS, RET}\times{RET}_{mat, bl}$ | (5) |
| $\frac{d{RBC}_{1}}{dt}=k_{LS, RET}\times{RET}_{mat, bl}-k_{LS, RBC}\times{RBC}_{1}$ | (6) |
| $\frac{d{RBC}_{2}}{dt}=k_{LS, RBC} \left( {RBC}_{1}-{RBC}_{2} \right)$ | (7) |
| $\frac{d{RBC}_{3}}{dt}=k_{LS, RBC} \left( {RBC}_{2}-{RBC}_{3} \right)$ | (8) |
| $\frac{d{RBC}_{4}}{dt}=k_{LS, RBC} \left( {RBC}_{3}-{RBC}_{4} \right)$ | (9) |
| $\frac{d{MCH}_{1}}{dt}=R_{in, MCH}-k_{LS, RBC}\times{MCH}_{1}$ | (10) |
| $\frac{d{MCH}_{2}}{dt}=k_{LS, RBC} \left( {MCH}_{1}-{MCH}_{2} \right)$ | (11) |
| $\frac{d{MCH}_{3}}{dt}=k_{LS, RBC} \left( {MCH}_{2}-{MCH}_{3} \right)$ | (12) |
| $\frac{d{MCH}_{4}}{dt}=k_{LS, RBC} \left( {MCH}_{3}-{MCH}_{4} \right)$ | (13) |
| $\frac{d{TOL}_{1}}{dt}= k_{TOL} \times PRE- k_{TOL}\times{TOL}_{1}$ | (14) |
| $\frac{d{TOL}_{2}}{dt}= k_{TOL} \times{TOL}_{1}- k_{TOL}\times{TOL}_{2}$ | (15) |

## 3. Algebraic derivation of parameterization for steady-state conditions

Assumptions

1. The system is in steady state (homeostasis) pre-dose.
2. Total maturation time from immature reticulocyte (compartment 2) to red blood cell (compartment 6) is the same no matter if it happens in the bone marrow or blood.

Definitions

Let:

- $i_{n}$ be the initial value of compartment *n*.
- ${RET}_{0}$ be the initial value of total reticulocyte count
- ${IRF}_{0}$ be the initial value of the immature reticulocyte fraction
- ${RBC}_{0}$ be the initial value of total erythrocyte count
- ${RBC}_{init}$ be the initial value of erythrocyte count in a single transit compartment
- ${LS}_{RBC}$ be the (estimated) mean lifespan of an erythrocyte
- $n_{CTR}$ be the number of transit compartments for erythrocytes ($n_{CTR}=4$)
- $k_{RBC}$ be the rate constant for erythrocyte transit, defined as: $k_{RBC}=\frac{n_{CTR}}{{LS}_{RBC}}$

Derivation

**Red Blood Cells in the First Transit Compartment (Compartment 6):** Since the total RBC count is distributed equally among *n*_CTR_ transit compartments, the initial value in each RBC compartment is:

$$i_{6}={RBC}_{init}=\frac{{RBC}_{0}}{n_{CTR}}$$

Immature reticulocytes in blood:

$$i_{4}={RET}_{0}\times{IRF}_{0}$$

Mature reticulocytes in blood:

$$i_{5}={RET}_{0}(1-{IRF}_{0})$$

Determining the rate constant *k*_RET_

At steady state, the rate of cells entering a compartment equals the rate of cells leaving it. For the transition from mature reticulocytes in blood (compartment 5) to erythrocytes (RBC) in blood (compartment 6):

$$k_{RET}{\times i}_{5}=k_{RBC}\times i_{6}$$

Solving for *k*_RET_:

$$k_{RET}\left( {RET}_{0}\times{IRF}_{0}+i_{3} \right)=k_{RET}\times{RET}_{0}\left( 1-{IRF}_{0} \right)\Rightarrow$$

$${RET}_{0}\times{IRF}_{0}+i_{3}={RET}_{0}\left( 1-{IRF}_{0} \right)\Rightarrow$$

Eq 1: Mature reticulocytes in bone marrow

$$i_{3}={RET}_{0}\left( 1-2\times{IRF}_{0} \right)$$

$$k_{RET}\times{RET}_{0}\times{IRF}_{0}=k_{RET}\times i_{2}\times p_{release}\Rightarrow$$

Eq 2: Immature reticulocytes in bone marrow

$$i_{2}=\frac{{RET}_{0}\times{IRF}_{0}}{p_{release}}$$

$$k_{RET}\times{RET}_{0}\left( 1-2\times{IRF}_{0} \right)=k_{RET}\times i_{2}\left( 1-p_{release} \right)\Rightarrow$$

$$\frac{{RET}_{0}\times{IRF}_{0}}{p_{release}}\left( 1-p_{release} \right)={RET}_{0}\left( 1-2\times{IRF}_{0} \right)\Rightarrow$$

$${\frac{{IRF}_{0}\left( 1-p_{release} \right)}{p_{release}}}=1-2\times{IRF}_{0}\Rightarrow$$

$$\frac{1-p_{release}}{p_{release}}=\frac{1}{{IRF}_{0}}-2\Rightarrow$$

$$1-p_{release}=p_{release}\left( \frac{1}{{IRF}_{0}}-2 \right)\Rightarrow$$

Eq 3: Probability of pre-mature release

$$p_{release}=-\frac{{IRF}_{0}}{{IRF}_{0}-1}={\frac{{IRF}_{0}}{1-{IRF}_{0}}}$$

## 4. NONMEM control stream of the model

$PROBLEM A semi-mechanistic population model to assess downstream drug-target effects on erythropoiesis

$INPUT

ID ; Subject identifier in this dataset

TIME ; Days after first dose

AMT ; Bitopertin dose amount

DV ; Dependent Variable

MDV ; Missing DV

BLQ ; Below Limit of Quantification

EVID ; Event ID

TYPE ; Type of observation

DOSE ; Dose given

ARM ; Treatment arm (bitopertin dose)

SEX ; 0=male, 1=female

AGE ; Subject age in years

ICL_BTP ; EBE from bitopertin PK model

$DATA data.csv IGNORE=@

;IGNORE(TYPE == 1) ; Bitopertin Dose

IGNORE(TYPE == 2) ; Bitopertin PK

IGNORE(TYPE == 3) ; CSF

;IGNORE(TYPE == 4) ; Reticulocyte Count

IGNORE(TYPE == 5) ; Reticulocyte MCH

;IGNORE(TYPE == 6) ; RBC Count

;IGNORE(TYPE == 7) ; HB

;IGNORE(TYPE == 8) ; RBC MCH

IGNORE(TYPE == 9) ; Soluble transferrin receptor

IGNORE(TYPE == 10) ; Ferritin

IGNORE(TYPE == 11) ; Iron

IGNORE(TYPE == 12) ; Transferrin

;IGNORE(TYPE == 13) ; Immature reticulocyte fraction

IGNORE(TYPE == 14) ; EPO

;IGNORE(TYPE == 15) ; Bitopertin AUC_24-hours

IGNORE(TYPE == 16)

IGNORE(TYPE == 17)

$SUBROUTINE ADVAN13 TOL=9

$ABBR PROTECT

$MODEL

COMP = (PRE) ; 1 - PRE

COMP = (PRE_RET1) ; 2 - Reticulocytes in bone marrow (immature)

COMP = (PRE_RET2) ; 3 - Reticulocytes in bone marrow

COMP = (RET1) ; 4 - Reticulocytes in blood (immature)

COMP = (RET2) ; 5 - Reticulocytes in blood

COMP = (RBC1) ; 6 - Transit compartment RBC

COMP = (RBC2) ; 7 - Transit compartment RBC

COMP = (RBC3) ; 8 - Transit compartment RBC

COMP = (RBC4) ; 9 - Transit compartment RBC

COMP = (MCH1) ; 10 - Transit compartment MCH

COMP = (MCH2) ; 11 - Transit compartment MCH

COMP = (MCH3) ; 12 - Transit compartment MCH

COMP = (MCH4) ; 13 - Transit compartment MCH

COMP = (M1) ; 14 - M1 - Moderator of RIN_PRE effect

COMP = (M2) ; 15 - M2 - Moderator of RIN_PRE effect

$INFN

IF (ICALL == 1) THEN

DOWHILE(DATA)

IF (TYPE == 7) MDV = 1 ; Total HB blood concentration

ENDDO

ENDIF

$PK

; Indicator variables ----------------------------------------------------------

placebo = 0

IF (DOSE == 0) placebo = 1

SEXF = 0

IF (SEX == 1) SEXF = 1

; TYPE -----------------------------------------------------------------------

dv_dose = 0

IF (TYPE == 1) dv_dose = 1

dv_pk = 0

IF (TYPE == 2) dv_pk = 1

dv_ret = 0

IF (TYPE == 4) dv_ret = 1

dv_rbc = 0

IF (TYPE == 6) dv_rbc = 1

dv_hb = 0

IF (TYPE == 7) dv_hb = 1

dv_mch = 0 ; rbc_hb

IF (TYPE == 8) dv_mch = 1

dv_ret_im = 0 ; Immature reticulocyte fraction

IF (TYPE == 13) dv_ret_im = 1

dv_epo = 0

IF (TYPE == 14) dv_epo = 1

dv_auc24 = 0

IF (TYPE == 15) dv_auc24 = 1

; Bitopertin PK --------------------------------------------------------------

F1 = 0 ; No drug into cmt1, drug modeled in NONMEM code

IF (NEWIND < 2) THEN

DRUG = 0

ADOS_BTP = 0

TDOS_BTP = 0

TAD_BTP = 0 ; Time after dose

TEND_BTP = 0

TREAT_BTP = 0

ENDIF

IF (EVID == 1) THEN

ADOS_BTP = AMT ; Actual last dose given

TDOS_BTP = TIME ; Time of last dose

TEND_BTP = TIME + 2 ; Two days after last dose

TAD_BTP = 0

TREAT_BTP = 1

ENDIF

IF (EVID /= 1 .AND. ADOS_BTP > 0) TAD_BTP = TIME - TDOS_BTP

IF (TIME > TEND_BTP) TREAT_BTP = 0

IF (placebo == 0) DRUG = ADOS_BTP / ICL_BTP * TREAT_BTP

; PD -------------------------------------------------------------------------

; Red blood cells (RBC)

TVLS_RBC = THETA(1)

MU_1 = LOG(TVLS_RBC)

LS_RBC = EXP(MU_1 + ETA(1)) ; RBC life span = MTT (mean transit time)

NCTR = 4 ; Number of RBC transit compartments

KLS_RBC = NCTR / LS_RBC ; k_tr, rate constant between transit compartments

TVRBC0 = (THETA(3) - THETA(10)**SEXF) * 1000

MU_3 = LOG(TVRBC0)

RBC0 = EXP(MU_3 + ETA(3))

; Immature reticulocyte fraction (IRF)

TVIRF0 = THETA(14)

MU_5 = LOG(TVIRF0)

IRF0 = EXP(MU_5 + ETA(5))

p_release = - IRF0 / (IRF0 - 1) ; Note: 0 < IRF0 < 0.5 for 0 < p_release < 1

; Reticulocytes (RET)

TVRET0 = THETA(11)

MU_4 = LOG(TVRET0)

RET0 = EXP(MU_4 + ETA(4))

KLS_RET = KLS_RBC * (RBC0 / NCTR) / (RET0 * (1 - IRF0))

LS_RET = 1 / KLS_RET ; Life span for mature reticulocytes in blood

LS_RET_MAT = 3 * LS_RET ; LS for entire RET lifespan from bone-marrow to RBC

; Precursors (PRE)

LS_PRE = 5

KLS_PRE = 1 / LS_PRE

RIN_PRE = KLS_RBC * (RBC0 / NCTR)

PRE0 = RIN_PRE / KLS_PRE

TVK_TOL = THETA(17)

MU_10 = LOG(TVK_TOL)

K_TOL = EXP(MU_10 + ETA(10))

; Mean corpuscular hemoglobin (MCH)

TVMCH0 = THETA(2)

MU_2 = LOG(TVMCH0)

MCH0 = EXP(MU_2 + ETA(2)) ; Hemoglobin (MCH) production rate

RIN_MCH = KLS_RBC * MCH0

; Hemoglobin (Hb)

HB0 = MCH0 * RBC0 ; Baseline HB

TVFDB = THETA(4)

MU_7 = LOG(TVFDB)

FDB = EXP(MU_7 + ETA(7))

; Bitopertin effect

TVIMAX_BTP = THETA(5)

MU_8 = LOG(TVIMAX_BTP)

IMAX_BTP = EXP(MU_8 + ETA(8))

TVIC50_BTP = THETA(6)

MU_6 = LOG(TVIC50_BTP)

AUC50_BTP = EXP(MU_6 + ETA(6))

TVGAM_BTP = THETA(7)

MU_9 = LOG(TVGAM_BTP)

GAM_BTP = EXP(MU_9 + ETA(9))

; Initial conditions ---------------------------------------------------------

A_0(1) = PRE0

A_0(14) = PRE0 ; Moderator 1

A_0(15) = PRE0 ; Moderator 2

A_0(2) = RET0 * (IRF0 / p_release)

A_0(3) = RET0 * (1 - 2 * IRF0)

A_0(4) = RET0 * IRF0 ; Immature reticulocytes

A_0(5) = RET0 * (1 - IRF0) ; Mature reticulocytes

RBC_init = RBC0 / NCTR

A_0(6) = RBC_init

A_0(7) = RBC_init

A_0(8) = RBC_init

A_0(9) = RBC_init

MCH_init = MCH0

A_0(10) = MCH_init

A_0(11) = MCH_init

A_0(12) = MCH_init

A_0(13) = MCH_init

$DES

; ------ PKPD ------

; Inhibition of HB synthesis rate in newly produced RBC

; (unobserved, before RBC release to the blood)

TRT_BTP = TREAT_BTP

IF (T > TEND_BTP) TRT_BTP = 0

INH_BTP = (1 - (TRT_BTP * (IMAX_BTP * DRUG**GAM_BTP) / (AUC50_BTP**GAM_BTP + DRUG**GAM_BTP)))

; Observed blood RBC, MCH, HB, and PRE

PR = A(1)

RT1 = A(2)

RT2 = A(3)

RT_IMM = A(4)

RT_MAT = A(5)

RT = RT_IMM + RT_MAT

RC1 = A(6)

RC2 = A(7)

RC3 = A(8)

RC4 = A(9)

RC = RC1 + RC2 + RC3 + RC4

MC1 = A(10)

MC2 = A(11)

MC3 = A(12)

MC4 = A(13)

HB1 = RC1 * MC1

HB2 = RC2 * MC2

HB3 = RC3 * MC3

HB4 = RC4 * MC4

HB = HB1 + HB2 + HB3 + HB4

MC = HB / RC

; System response: Stimulation of PRE production proportional to Hb decrease

HB_CFB = (HB0 - HB) / HB0

HB_STIM_PRE = EXP(HB_CFB * FDB)

HB_STIM_IRF = EXP(HB_CFB * FDB)

; ------ Precursors ------

DADT(1) = RIN_PRE * HB_STIM_PRE * (PRE0 / A(15)) - KLS_PRE * PR

DADT(14) = K_TOL * A(1) - K_TOL * A(14)

DADT(15) = K_TOL * A(14) - K_TOL * A(15)

; ------ Reticulocytes in bone marrow (immature) ------

DADT(2) = KLS_PRE * PR - KLS_RET * HB_STIM_IRF * (RT1 * p_release) - KLS_RET * (RT1 * (1 - p_release))

; ------ Reticulocytes in bone marrow (mature) ------

DADT(3) = KLS_RET * (RT1 * (1 - p_release)) - KLS_RET * HB_STIM_IRF * RT2

; ------ Reticulocytes in blood (immature) ------

DADT(4) = KLS_RET * HB_STIM_IRF * (RT1 * p_release) - KLS_RET * RT_IMM

; ------ Reticulocytes in blood (mature) ------

DADT(5) = KLS_RET * HB_STIM_IRF * RT2 + KLS_RET * RT_IMM - KLS_RET * RT_MAT

; ------ Red blood cells ------

DADT(6) = KLS_RET * RT_MAT - KLS_RBC * RC1

DADT(7) = KLS_RBC * (RC1 - RC2)

DADT(8) = KLS_RBC * (RC2 - RC3)

DADT(9) = KLS_RBC * (RC3 - RC4)

; -------- MCH --------

DADT(10) = RIN_MCH * INH_BTP - KLS_RBC * MC1

DADT(11) = KLS_RBC * (MC1 - MC2)

DADT(12) = KLS_RBC * (MC2 - MC3)

DADT(13) = KLS_RBC * (MC3 - MC4)

$ERROR

; -------- PD --------

PRE = A(1)

RET1 = A(2)

RET2 = A(3)

RET_IMM = A(4)

RET_MAT = A(5)

RET = RET_IMM + RET_MAT

RBC1 = A(6)

RBC2 = A(7)

RBC3 = A(8)

RBC4 = A(9)

RBC = RBC1 + RBC2 + RBC3 + RBC4

MCH1 = A(10)

MCH2 = A(11)

MCH3 = A(12)

MCH4 = A(13)

HMG1 = RBC1 * MCH1

HMG2 = RBC2 * MCH2

HMG3 = RBC3 * MCH3

HMG4 = RBC4 * MCH4

HMG = HMG1 + HMG2 + HMG3 + HMG4

MCH = HMG / RBC

IRF = RET_IMM / RET

; -------- IRF --------

IF (dv_ret_im == 1) THEN

IPRED = IRF ; 10^9

W = SQRT((THETA(16)*IPRED)**2 + THETA(15)**2)

ENDIF

; -------- RET --------

IF (dv_ret == 1) THEN

IPRED = RET ; 10^9

W = SQRT((THETA(12)*IPRED)**2 + THETA(13)**2)

ENDIF

;-------- RBC --------

IF (dv_rbc == 1) THEN

IPRED = RBC / 1000 ; convert 10^9 to 10^12/L

W = THETA(8)

ENDIF

; -------- MCH --------

IF (dv_mch == 1) THEN

IPRED = MCH

W = THETA(9)

ENDIF

; -------- HB --------

IF (dv_hb == 1) THEN

IPRED = HMG / 1000

W = THETA(8) + THETA(9)

ENDIF

IRES = DV - IPRED

IWRES = IRES / W

Y = IPRED + W * EPS(1)

$THETA

(1,124,200) ; 1 LS_RBC

(0,29.8) ; 2 TVMCH0

(0,5.93) ; 3 TVRBC0 males (subtract 1 unit to account for parameterisation)

10 ; 4 TVFDB

(0,0.6,1) FIX ; 5 IMAX_BTP

(0,16.3) ; 6 AUC50_BTP

1 FIX ; 7 GAM_BTP

(0,0.173) ; 8 Add_err_RBC (%CV)

(0,0.346) ; 9 Add_err_MCH (%CV)

1.59 ; 10 TVRBC0 females

(0,39.9) ; 11 TVRET0

(0,0.217) ; 12 Prop_err_RET (%CV)

0 FIX ; 13 Add_err_RET

(0,0.045,0.5) ; 14 IRF0

(0,0.00996) ; 15 Add_err_IRF

(0,0.37) ; 16 Prop_err_IRF

(0,0.0234) ; 17 TVK_TOL

$OMEGA BLOCK(6)

0.0578 ; 1 IIV LS_RBC

0.000114545187589877 0.00227 ; 2 IIV MCH0

0.000127669886817526 2.53009881229963E-05 0.00282 ; 3 IIV RBC0

0.00061670414300538 0.000122215383647068 0.000136218941414181 0.0658 ; 4 IIV RET0

0.000756070102569861 0.000149834241747339 0.000167002395192404 0.000806698208749716 0.0989 ; 5 IIV IRF0

0.00165869828480046 0.00032871264046276 0.000366376855164187 0.00176976834642277 0.00216970965799574 0.476 ; 6 IIV AUC50_BTP

$OMEGA

0.0225 FIX ; 7 IIV FDB

0.0225 FIX ; 8 IIV IMAX_BTP

0.0225 FIX ; 9 IIV GAM_BTP

0.0225 FIX ; 10 IIV K_TOL

$SIGMA 1 FIX

$ESTIMATION METHOD=SAEM GRD=TS(8,9,12,13,15,16) PRINT=1 NOCOV=0 INTERACTION NITER=400 AUTO=1 SEED=1337 RANMETHOD=3S2P NSIG=3 SIGL=9

$ESTIMATION METHOD=IMP GRD=TS(8,9,12,13,15,16) PRINT=1 NOCOV=0 ISAMPLE=3000 NITER=5 AUTO=0 SEED=1337 RANMETHOD=3S2P MAPITER=0 EONLY=1 NSIG=3 SIGL=9 MSFO=run1.msf

$COVARIANCE PRINT=E UNCONDITIONAL MATRIX=R
